# Ginseng Pectin WGPA Alleviates Exercise-Induced Fatigue by Enhancing Gluconeogenesis

**DOI:** 10.1155/2022/7973380

**Published:** 2022-12-16

**Authors:** Yang Jiao, Juanjuan Chen, Fengqi Hao, Meihong Tian

**Affiliations:** ^1^School of Physical Education, Northeast Normal University, 5268 Renmin Street, Changchun, Jilin 130024, China; ^2^Key Laboratory of Molecular Epigenetics of the Ministry of Education (MOE), Northeast Normal University, 5268 Renmin Street, Changchun, Jilin 130024, China

## Abstract

With the development of medicine and sport science, growing attention has been paid to the recovery of exercise-induced fatigue. Ginseng pectin has been shown to be important for a variety of biological functions. Although many studies suggest that ginseng pectin plays an important role in the alleviation of exercise-induced fatigue, the underlying mechanism still remains unclear. In this study, C57BL/6J mice were subjected to a wheel apparatus for exhaustive exercise and fed with ginseng pectin WGPA (acidic fraction of water-soluble ginseng polysaccharides) afterwards. Subsequently, a series of physiological and biochemical indexes, such as blood lactic acid, blood glucose, muscle glycogen, insulin, and glucagon, is evaluated. Meanwhile, enzymatic activity and mRNA level of key enzymes involved in hepatic gluconeogenesis are analyzed. Our results demonstrate that the treatment of ginseng pectin WGPA can result in enhanced gluconeogenesis and decreased insulin and in turn facilitate the recovery of exercise-induced fatigue. In response to WGPA treatment, both phosphoenolpyruvate carboxykinase (PEPCK) and glucose 6 phosphatase (G6Pase) activity were upregulated, indicating that these two enzymes play a critical role in WGPA-induced upregulation in gluconeogenesis. Moreover, mRNA level of G6Pase, but not PEPCK, was increased upon WGPA treatment, suggesting that G6Pase expression is regulated by WGPA. Importantly, the presence of WGPA downregulated insulin both *in vivo* and *in vitro*, suggesting the upregulation in gluconeogenesis may be due to alterations in insulin. Together, we provide evidence that ginseng pectin WGPA is able to alleviate exercise-induced fatigue by reducing insulin and enhancing gluconeogenesis.

## 1. Introduction

How to alleviate exercise-induced fatigue and facilitate fatigue recovery is a key challenge in the field of exercise science and sports [1–4]. Efficient elimination of exercise-induced fatigue can greatly improve physical function and competition ability for athletes [5–8]. Obviously, inadequate training is unlikely to yield satisfactory achievement, while overtraining can directly cause physical fatigue [2, 9]. To obtain ideal achievements in competitive sports, athletes often dedicate themselves to intense and extensive training, which can cause severe exercise-induced fatigue and thereby affect progression in professional training [2, 3, 9]. Characterization of mechanism involved in exercise-induced fatigue and fatigue recovery will help us explore effective strategies to improve training efficiency.

Exercise-induced fatigue is mainly caused by the depletion of energy source [1]. It is usually temporary and prone to cause compromise in physical training but can be recovered with time [1, 3, 6]. During professional training when athletes can go through multiple rounds of “fatigue-recovery,” timely nutrient supply is critical for an efficient fatigue recovery [7, 10].

When nutritional supply is low in liver, gluconeogenesis and glycogenolysis are upregulated to maintain normoglycemic state [11]. In particular, gluconeogenesis, which accounts for approximately half of the blood glucose and energy supply for major organs, needs to be enhanced to facilitate the conversion of lactate, glycerol, and glycogenic amino acids into glucose for blood glucose and glycogen regeneration [12]. Although glucose synthesis initiated from pyruvate seems similar to reversed glycolysis, gluconeogenesis chooses four alternative reactions driven by pyruvate carboxylase (PC), phosphoenolpyruvate carboxykinase (PEPCK), fructose1, 6-bisphosphatase (FBPase), and glucose 6 phosphatase (G6Pase) to avoid reversing reactions controlled by hexokinase (HK), phosphofructokinase (PFK), and pyruvate kinase (PK) in glycolysis [12, 13]. Meanwhile, lactate dehydrogenase (LDH) catalyzes lactate into pyruvate, which is subsequently used in TCA cycle or gluconeogenesis [14]. Therefore, key enzymes in gluconeogenesis as well as LDH are critical for the recovery of blood sugar level after intense physical training.

In recent years, improvements in isolation and purification technology have greatly advanced structural and functional characterization for effective ingredients in ginseng [15, 16]. Basically, ginseng polysaccharides can be categorized into neutral polysaccharide and acidic polysaccharide [16]. Using diethylaminoethyl (DEAE)-cellulose ion-exchange chromatography, ginseng water soluble polysaccharide (WGP) can be separated into unbound fraction (WGPN) and acidic fraction (WGPA). Ginseng pectin is acidic polysaccharide and consists of galacturonic acid (GalA), galactose (Gal), arabinose (Ara), and rhamnose (Rha) [16]. Mounting evidence suggests that ginseng polysaccharides have multiple functions, including immune rebalancing, hypoglycemic maintenance, and prevention of tumor development [17–19]. However, whether ginseng pectin can relieve exercise-induced fatigue still remains unclear.

## 2. Materials and Methods

### 2.1. Animals

Male C57BL/6J mice (10- to 11 wk old, 27.53 ± 2.328 g) were purchased from Beijing HFK Bioscience Co., Ltd, China and bred in a specific pathogen-free facility with a standard 12 hr alternate light/dark cycle at an ambient temperature of 22 ± 2°C and 30%–70% humidity at the Animal Research Center of Northeast Normal University (Changchun, China). All mouse experiments were conducted in accordance with the protocols for animal use, treatment, and euthanasia approved by the Animal Care Committee of Northeast Normal University.

### 2.2. Ginseng Acidic Polysaccharide Extraction

Water soluble ginseng polysaccharide' s acidic fraction (WGPA) was provided by Prof. Y. F. Zhou (School of Life Sciences, Northeast Normal University, China). The preparation of WGPA was performed as described previously [16]. In brief, ginseng roots were extracted with appropriate distilled water at 100°C for 4 h and filtered through four sheets of gauze. The solid material was extracted twice again under the same conditions. All filtrates were collected and centrifuged to remove water-insoluble materials followed by precipitation by the addition of 95% ethanol. After centrifugation, the precipitate was dried by solvent exchange for the preparation of crude ginseng polysaccharides, which were redissolved in distilled water and treated with Sevag reagent to remove proteins. The deproteined water soluble polysaccharide (WGP) fraction was loaded on a diethylaminoethyl (DEAE)-cellulose column and was eluted with distilled water to remove the unbound fraction (WGPN) and then with 0.5 M NaCl to elute the WGPA.

### 2.3. Culturing of Min6 Cell Line

Mouse pancreatic *β*-cell line Min6 was regularly maintained in Dulbecco's modified Eagle medium (DMEM) (Sigma) supplemented with 15% FBS (Biological Industries), 1% penicillin and streptomycin and 50 *μ*M *β*-mercaptoethanol (PAN Biotech). The cells were maintained at 37°C in a humidified incubator with 5% CO_2_.

### 2.4. Insulin Secretion Assay

The assessment of insulin secretion was performed according to the previously described protocol [20, 21] with modifications. Min6 cells were incubated in 48-well plates (5 × 10^4^ cells per well) for 48 h in maintenance medium and gently washed twice with Krebs−Ringer bicarbonate‐HEPES (KRBH) buffer of pH 7.4 containing NaCl (115 mM), KCl (4.7 mM), MgSO_4_·7 H_2_O (1.2 mM), KH_2_PO_4_ (1.2 mM), NaHCO_3_ (20 mM), HEPES (16 mM), CaCl_2_ (2.56 mM), and BSA (0.2% w/v). Min6 cells were then incubated in KRBH buffer supplemented with 25 mM glucose, and 15 mM sodium lactate as a mimic for exercise-induced increased lactic acid. After stimulation with ginseng pectin WGPA dissolved in KRBH buffer for 1 h, the insulin level in the supernatants were detected and estimated with the Mouse Insulin ELISA Kit (Nanjing Jiancheng Bioengineering Institute, China) following the manufacturer's instructions.

### 2.5. Wheel Running Exercise-Induced Fatigue and WGPA Administration

The exercise-induced fatigue mouse model was established using Rotary Fatigue Tester for Mice (YLS-10B, Jinan Yiyan Science & Technology Development Co., Ltd). All mice were allowed to acclimatize themselves to the exercise for one week (adaptation period). The parameters of exercise training in adaptation period include the following: first day, 10 r/min for 10 minutes; second day, having a rest; third day, 15 r/min for 10 minutes prior to 20 r/min for 5 minutes, followed by incremental 5 r/min to 45 r/min for 5 minutes; fourth day, having a rest; fifth day, repeat third day's training prior to two days' rest. After this period, mice were subject to exercise-induced fatigue with 30 r/min. While exercising, mice were stimulated by gentle electrical stimulation with 2 mA pulse for 5 s. Mice bearing 30 s of electrical impulse combined with hyperpnea, depression, and slow reaction to capture were recognized as fatigue. The exhausted mice were administrated with WGPA (200 mg/kg) dissolved in physiological saline by oral gavage (300 *μ*l) and supplied with 300 *μ*l physiological saline (0.9% NaCl) and were included as negative control prior to 3 h resting, which allowed free access to drinking without food.

### 2.6. Analysis of Biochemical Parameters in Serum

After determination of body weight, serum samples of mice were prepared for the assessment of level of lactic acid (LA), malonaldehyde (MDA), blood glucose, blood urea nitrogen (BUN), insulin, and glucagon, as well as the activity of superoxide dismutase (SOD), lactate dehydrogenase (LDH), and creatine phosphokinase (CK). All the parameters were determined using commercially available kits (Nanjing Jiancheng Bioengineering Institute, China) according to the manufacturer's recommended instructions.

### 2.7. Assessment of Biochemical Indexes in Liver and Muscle

The muscle and hepatic glycogen level was evaluated by glycogen assay kit (Nanjing Jiancheng Bioengineering Institute, China). The activity of LDH was assayed using commercial kit purchased from Nanjing Jiancheng Bioengineering Institute (China), as well as enzymatic activity assay kits which used to determine the activity of pyruvate carboxylase (PC), phosphoenolpyruvate carboxykinase (PEPCK), fructose-1,6-bisphosphate phosphatase (FBPase), and glucose-6-phosphatase (G6Pase) were provided by Suzhou Comin Biotechnology Co., Ltd (China). All the assessments of abovementioned biochemical indicators were performed according to the kit's protocol.

### 2.8. Real-Time PCR

Total RNA was isolated from liver tissue using the TRIzol reagent (Invitrogen). cDNA was synthesized with the Trans Script One-Step gDNA Removal and cDNA Synthesis Super Mix (TransGenBiotech, China) according to manufacturer's instructions. Real-time PCR was performed on the QuantStudio 3 real-time PCR instrument (Applied Biosystems) with a SYBR Premix Ex Taq (Tli RNase H Plus) regent (Takara). The mRNA expression of genes was normalized to the expression of *β*-actin gene. Data were analyzed using the comparative cycling threshold method. Primer sequences were listed as follows: 5′-TGGTAGCCCTGTCTTTCTTTG-3′ and 5′-TTCCAGCATTCACACTTTCCT-3′ for *G6Pase*; 5′-ACACACACACATGCTCACAC-3′ and 5′-ATCACCGCATAGTCTCTGAA-3′ for *PEPCK*; 5′-GGCTGTATTCCCCTCCATCG-3′ and 5′-CCAGTTGGTAACAATGCCATGT-3′ for *Actb*.

### 2.9. Data Analysis and Statistics

Statistical analysis was performed using GraphPad Prism version 6 (GraphPad Software). Data are displayed as mean ± standard (SD). The P values were calculated from student unpaired *t*-test or one-way ANOVA test. *P* values < 0.05 were considered significant. *P* values were indicated on graphs as *P* < 0.05^*∗*^, *P* < 0.01^*∗∗*^, *P* < 0.001^*∗∗∗*^.

## 3. Results

**Figure 1:**
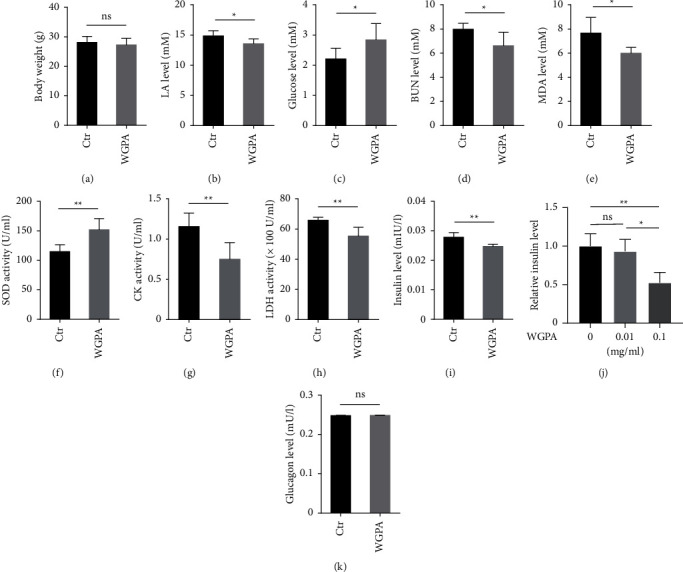
Effect of WGPA on biochemical parameters in serum from exercise-induced fatigue mice. 10- to 11-wk -old male C57BL/6J mice were subject to the established exercise-induced fatigue model using Rotary Fatigue Tester. Experimental and technical details were included in “Materials and Methods.” The exhausted mice were administrated with WGPA prior to 3 h resting. After determination of body weight (a), serum was collected for the assessment of lactic acid (LA) (b), blood glucose (c), blood urea nitrogen (BUN) (d) and malonaldehyde (MDA) (e) level. The activity of superoxide dismutase (SOD) (f), creatine phosphokinase (CK) (g) and lactate dehydrogenase (LDH) (h), as well as the level of insulin (i) and glucagon (k) in serum were evaluated. (j) Ginseng pectin WGPA downregulates insulin secretion from Min6 cells. Min6 cells were incubated in KRBH buffer supplemented with 25 mM glucose and 15 mM sodium lactate. At the onset of incubation, WGPA was added into the medium with indicated concentrations. After 1 h stimulation, the supernatants were collected for insulin assessment. (a)–(i) and (k) Data represent mean ± SD (*n* = 6) with significance determined by student unpaired *t*-test. (j) Data represent mean ± SD (*n* = 3) with significance determined by one-way ANOVA test. *P* < 0.05^*∗*^; *P* < 0.01^*∗∗*^; ns, nonsignificant.

To evaluate the role of ginseng pectin WGPA in exercise-induced recovery, we set out to analyze a series of physiological and biochemical parameters to gain insights into fatigue recovery as well as underlying mechanism. After exhaustive exercise, mice fed with 0.9% NaCl (control group) or ginseng WGPA (WGPA group) were weighed immediately. Means for control group and WGPA group were 27.26 ± 2.242 g and 28.09 ± 2.055 g, respectively (Figure 1(a)). There was no significant change between these two groups. Next, both control and WGPA-treated mice were subjected to systematic analysis for a series of biochemical parameters. We found that WGPA-treated mice exhibited a significant downregulation in lactic acid (Figure 1(b)) and enhancement in the glucose level (Figure 1(c)) from blood as compared to the control group.

Given that BUN is linked with protein and amino acid catabolism and its upregulation is associated with energy exhaustion and overloaded training as well as delayed recovery [22, 23], we measured this parameter and observed a decrease upon WGPA treatment (Figure 1(d)). Moreover, we examined the level of MDA and SOD. MDA is the final product of oxidative reaction of membrane lipid and its upregulation reflects structural alterations or functional defects in membranes [24]. SOD activity is directly relevant to the clearance of reactive oxygen species (ROS) and often analyzed along with MDA for the evaluation of cell damage caused by ROS [25]. In response to WGPA treatment, MDA was downregulated (Figure 1(e)), while SOD was upregulated (Figure 1(f)). These changes argue an important role for WGPA in fatigue recovery. In addition, CK and LDH activities are typically used for the evaluation of damages in skeletal muscle and cell membrane, respectively [26]. In our analysis, both CK and LDH activities in serum were decreased after WGPA treatment (Figures 1(g) and 1(h)). Meanwhile, the level of insulin, but not glucagon, dropped in WGPA-treated mice (Figures 1(i) and 1(k)). In line with this observation, the presence of WGPA downregulated the level of insulin secreted by murine pancreatic *β*-cell, Min6 (Figure 1(j)), which is a classic cell model widely used for evaluating insulin secretion and function in vitro [20, 21, 27–30].

**Figure 2:**
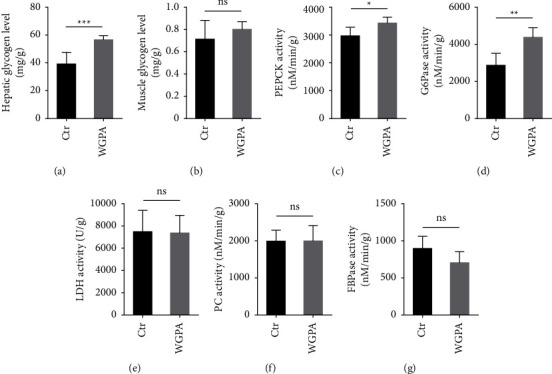
Impact from WGPA on biochemical indexes in liver and muscle. Mice were subject to exhausted exercise and WGPA administration as described in Figure 1(a). Hepatic glycogen level (a), the activity of phosphoenolpyruvate carboxykinase (PEPCK) (c), glucose-6-phosphatase (G6Pase) (d), lactate dehydrogenase (LDH) (e), pyruvate carboxylase (PC) (f) and fructose-1,6-bisphosphate phosphatase (FBPase) (g) were detected in liver tissues. Gastrocnemius muscles were isolated from mice for the detection of muscle glycogen (b). Data represent mean ± SD (*n* = 6) with significance determined by student unpaired *t*-test. *P* < 0.05^*∗*^; *P* < 0.01^*∗∗*^; *P* < 0.001^*∗∗∗*^; ns, nonsignificant.

**Figure 3:**
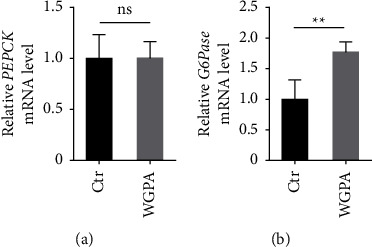
WGPA enhances *G6Pase* expression in liver tissues. Total RNA was isolated form liver tissues of mice treated as described in Figure 1. After cDNA synthesized, the mRNA level of *PEPCK* (a) and *G6Pase* (b) was determined by RT-qPCR. Data represent mean ± SD (*n* = 6) with significance determined by student unpaired *t*-test. *P* < 0.01^*∗∗*^; ns, nonsignificant.

In parallel, we examined a set of biochemical characteristics in liver and muscle to dissect the impact from WGPA on fatigue recovery. Interestingly, the level of hepatic glycogen was higher in WGPA-treated mice as compared to control mice (Figure 2(a)). In contrast, the levels of muscle glycogen in different groups seemed to be rather similar (Figure 2(b)). Moreover, while both PEPCK and G6Pase activities in liver were upregulated upon WGPA treatment (Figures 2(c) and 2(d)), and LDH, PC, and FBPase activities appeared to be rather stable regardless of WGPA treatment (Figures 2(e)–2(g)). Because mRNA is a direct readout for gene expression, we further analyzed PEPCK and G6Pase mRNA level in the presence or absence of WGPA. The mRNA level of PEPCK remained largely unaffected, while the G6Pase mRNA level increased significantly upon WGPA treatment (Figures 3(a) and 3(b)).

## 4. Discussion

In this work, mice were exhausted through wheel training instead of swimming and running on a platform. Wheel training can provide a continuous running opportunity, which fulfills the climbing-running nature of mice and avoids spontaneous stress from forced swimming. Among physiological and biochemical parameters that we examined, the lactate level in blood was effectively reduced by WGPA treatment. Lactate is the final product of glycolysis and directly contributes to the accumulation of H^+^ [14, 31]. Declined pH in muscle and blood leads to disturbed metabolic homeostasis in the body and dampened signal transfer at neuron-muscle connection [14, 31]. A simultaneous decrease in Ca^2+^ concentration disrupts the interaction between myosin and actin, resulting in impaired muscle function eventually 1. Given that lactate is generated via glycolysis and removed by gluconeogenesis [12, 14], the observation that WGPA promoted the removal of lactate suggests that WGPA has an impact on both metabolic pathways.

Under normal condition, the nitrogen level in the body is maintained at a proper level and the generation and release of BUN are well balanced [32]. BUN is an important final product from protein metabolism and its upregulation is usually associated with deregulated renal function [32]. When the body experiences exercise-induced fatigue, energy sources, including glucose and fatty acid, burn up rapidly [14, 33]. Meanwhile, protein catabolism is improved and BUN is upregulated [24, 32]. WGPA treatment significantly lowered down BUN, suggesting that WGPA play a role in the regulation of renal function. Continuous and exhaustive training usually leads to enhanced oxidative metabolism and accumulation of ROS in skeletal muscle [26, 34, 35]. It is known that ROS can target different biological macromolecules, including sugar, lipid, protein, and nucleic acid and thereby causes destructive effect on cell membrane or mitochondrial membrane [26, 34, 35]. MDA is the final product of oxidative reaction of membrane lipid and its upregulation reflects structural alterations or functional defects in membranes [24, 36]. SOD activity is directly relevant to the clearance of ROS and often analyzed along with MDA for the evaluation of cell damage caused by ROS [24, 35]. CK and LDH activities in serum are typically used for the evaluation of damage in skeletal muscle and cell membrane, respectively [26, 32]. Based on changes in MDA, CK, SOD, and LDH, we propose that WGPA is capable of promoting the removal of ROS and limiting the generation of damage in cells. In addition, our data showed that WGPA significantly decreased the level of insulin rather than glucagon in mice that went through intense physical training, suggesting a balanced metabolic status among sugar, protein, and fatty acid after WGPA treatment. Together, these observations argue that WGPA can promote the recovery of exercise-induced fatigue probably by accelerating the clearance of metabolic waste and facilitating damage repair in muscles.

Glucose is the main source responsible for energy production and stored as glycogen in liver and muscle. When the level of blood glucose drops due to rapid consumption of glycogens by muscle during persistent and intense training, hepatic glycogens are hydrolized to maintain a proper level of blood glucose [37]. Our data revealed that WGPA facilitated the recovery of hepatic glycogen and blood glucose rather than muscle glycogen. This is in agreement with long period of time, which can be up to 46 hours, and is needed for the regeneration of muscle glycogens. Liver is an important organ providing approximately 80% of the total glucose through hydrolysis of hepatic glycogen as well as gluconeogenesis and plays a central role in the homeostasis maintenance of blood glucose [37]. During fasting, gluconeogenesis is enhanced to improve glucose yield, which accounts for 70% of the total glucose needed by the body after 24 h-fasting and 90% after 48 h-fasting [37]. In exhausted mice deprived for food, WGPA promoted the recovery of blood glucose and hepatic glycogen, indicating an important role for WGPA in gluconeogenesis and insulin secretion and thereby recovery from exercise-induced fatigue.

Besides PC, PEPCK, FBPase, and G6Pase, the four rate-liming enzymes directly involved in gluconeogenesis [12, 38] and LDH that converts lactate into pyruvate are also critical for gluconeogenesis [31]. In response to WGPA treatment, PEPCK and G6Pase activities were enhanced.

It is known that diabetic patients with impaired glucose tolerance are often compromised in insulin secretion [39, 40]. It seems that ginseng treatment that appears to associate with reduced insulin and enhanced gluconeogenesis might worsen the entire situation for them. However, impact from ginseng on different individuals may vary due to different physiological conditions and metabolic status [41]. In our study, we focus on the recovery of exercise-induced fatigue where metabolic status is most likely different from an unperturbed condition. The observation that ginseng pectin WGPA induced an upregulation in blood glucose and a downregulation in insulin during fatigue recovery still needs to be corroborated carefully under a normal condition in future. Nevertheless, ginseng treatment for people with diabetes or those with impaired glucose tolerance probably needs to be carefully evaluated.

## 5. Conclusions

Efficient elimination of exercise-induced fatigue can greatly improve physical function and competition ability for athletes. It has been suggested that ginseng pectin plays an important role in the alleviation of exercise-induced fatigue. However, the underlying mechanism still remains elusive. We found that ginseng pectin WGPA can upregulate the activity of both PEPCK and G6Pase, two key enzymes in gluconeogenesis, and decrease insulin level in plasma, in the facilitation of exercise-induced fatigue recovery. Moreover, mRNA expression of G6Pase, but not PEPCK, was enhanced by ginseng pectin WGPA. Taken together, our findings depict a previously unappreciated role for the recovery of exercise-induced fatigue by ginseng pectin, suggesting that ginseng pectin could be a reference for the development of healthy food supplements for athletes.

## Data Availability

The data used to support the findings of this study are available from the corresponding authors upon request.
